# Supplementary material for: Molecular and Functional Relevance of NaV1.8-Induced Atrial Arrhythmogenic Triggers in a Human SCN10A Knock-Out Stem Cell Model
Source: Int J Mol Sci. 2023 Jun 15;24(12):10189. doi: 10.3390/ijms241210189 (PMC10299073; doi:10.3390/ijms241210189)
Supplement: Supplementary file 1 [file ijms-24-10189-s001.zip › ijms-2404701-supplementary.pdf]

Molecular and functional relevance of Na_V1.8-induced atrial arrhythmogenic triggers in a human *SCN10A* knock out stem cell model

Nico Hartmann, Maria Knierim, Wiebke Maurer, Nataliya Dybkova, Gerd Hasenfuß, Samuel Sossalla, Katrin Streckfuss-Bömeke

Supplementary Materials

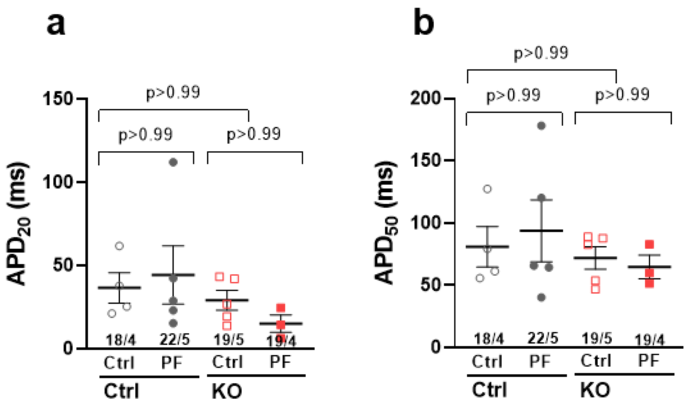

Figure S1. *The following figures are all shown at* **1 Hz**. *(**a**)Mean data (nested) ± SEM of APD20 (atrial control n=18 cells/4 differentiations; atrial control + PF n=22 cells/5 differentiations; SCN10A KO control n=19 cells/5 differentiations, SCN10A KO + PF n=19 cells/4 differentiations); (**b**) Mean data (nested) ± SEM of APD50 (atrial control n=18 cells/4 differentiations; atrial control + PF n=22 cells/5 differentiations; SCN10A KO control n=19 cells/5 differentiations, SCN10A KO + PF n=19 cells/4 differentiations) and (**c**) Mean data (nested) ± SEM of APD70 (atrial control n=18 cells/4 differentiations; atrial control + PF n=22 cells/5 differentiations; SCN10A KO control n=19 cells/5 differentiations, SCN10A KO + PF n=19 cells/4 differentiations); statistics with nested 1 way ANOVA.*

Table S1. *Action potential duration of atrial iPSC-CM (APD20, APD50 and APD70) at* **1 Hz**. *(**left**) Mean data (nested) ± SEM of APD20 (atrial control n=18 cells/4 differentiations; atrial control + PF n=22 cells/5 differentiations; SCN10A KO control n=19 cells/5 differentiations, SCN10A KO + PF n=19 cells/4 differentiations). (**middle**) Mean data (nested) ± SEM of APD50 (atrial control n=18 cells/4 differentiations; atrial control + PF n=22 cells/5 differentiations; SCN10A KO control n=19 cells/5 differentiations, SCN10A KO + PF n=19 cells/4 differentiations) and (**right**) Mean data (nested) ± SEM of APD70 (atrial control n=18 cells/4 differentiations; atrial control + PF n=22 cells/5 differentiations; SCN10A KO control n=19 cells/5 differentiations, SCN10A KO + PF n=19 cells/4 differentiations); statistics with nested 1 way ANOVA.*

	**APD_{20}**	**APD_{50}**	**APD_{70}**
atrial control	34.2±7.9	77.2±13.1	109.8±15.9
atrial control + PF	35.1±7.6	82.3±12.4	118.9±14.5
SCN10A KO control	32.0±7.0	72.0±12.5	109.8±17.2
SCN10A KO + PF	14.5±5.7	63.7±15.7	90.1±18.2
